# Inexpensive Home Infrared Living/Environment Sensor with Regional Thermal Information for Infant Physical and Psychological Development

**DOI:** 10.3390/ijerph17186844

**Published:** 2020-09-19

**Authors:** Genta Karino, Aya Senoo, Tetsuya Kunikata, Yoshimasa Kamei, Hideo Yamanouchi, Shun Nakamura, Masanori Shukuya, Ricki J. Colman, Mamiko Koshiba

**Affiliations:** 1 Graduate School of Engineering, Tokyo University of Agriculture and Technology, Tokyo 184-8588, Japan; gentachan2011@gmail.com (G.K.); fa52842@fa2.so-net.ne.jp (A.S.); nakashn@cc.tuat.ac.jp (S.N.); 2 Department of Pediatrics, Saitama Medical University, Saitama 350-0495, Japan; tekunika@saitama-med.ac.jp (T.K.); yhideo@saitama-med.ac.jp (H.Y.); 3 Research Fellow of the Japan Society for the Promotion of Science, Tokyo 102-0083, Japan; 4 Department of Obstetrics and Gynecology, Saitama Medical University, Saitama 350-0495, Japan; ykamei1019@gmail.com; 5 Faculty of Environmental Studies Department of Restoration Ecology and Built Environment, Tokyo City University, Kanagawa 224-8551, Japan; shukuya@tcu.ac.jp; 6 Wisconsin National Primate Research Center, University of Wisconsin, Madison, WI 53715, USA; 7 Department of Cell & Regenerative Biology, School of Medicine and Public Health, University of Wisconsin, Madison, WI 53705, USA; 8 Graduate School of Science and Technology for Innovation, Yamaguchi University, Yamaguchi 755-8611, Japan; 9 Graduate School of Information Sciences, Tohoku University, Sendai 980-8579, Japan

**Keywords:** infrared image sensor, life monitoring, primate model, common marmoset, infant development, complex change of circadian rhythms, locomotion, body temperature, feeding influence

## Abstract

The use of home-based image sensors for biological and environmental monitoring provides novel insight into health and development but it is difficult to evaluate people during their normal activities in their home. Therefore, we developed a low-cost infrared (IR) technology-based motion, location, temperature and thermal environment detection system that can be used non-invasively for long-term studies in the home environment. We tested this technology along with the associated analysis algorithm to visualize the effects of parental care and thermal environment on developmental state change in a non-human primate model, the common marmoset (*Callithrix jacchus*). To validate this system, we first compared it to a manual analysis technique and we then assessed the development of circadian rhythms in common marmosets from postnatal day 15–45. The semi-automatically tracked biological indices of locomotion velocity (BV) and body surface temperature (BT) and the potential psychological index of place preference toward the door (BD), showed age-dependent shifts in circadian phase patterns. Although environmental variables appeared to affect circadian rhythm development, principal component analysis and signal superimposing imaging methods revealed a novel phasic pattern of BD-BT correlation day/night switching in animals older than postnatal day 38 (approximately equivalent to one year of age in humans). The origin of this switch was related to earlier development of body temperature (BT) rhythms and alteration of psychological behavior rhythms (BD) around earlier feeding times. We propose that this cost-effective, inclusive sensing and analytic technique has value for understanding developmental care conditions for which continual home non-invasive monitoring would be beneficial and further suggest the potential to adapt this technique for use in humans.

## 1. Introduction

Recent improvements in image sensors enable us to use them for novel human detection [1,2]. A sensor with high sensitivity and resolution may have advanced possibilities but is also expensive and requires a powerful signal-processing central processing unit (CPU). Alternatively, an inexpensive and low resolution infrared image sensor life monitoring application requiring a lighter image processing load, opens the possibility of longitudinal, home-based assessments of biological data that can inform understanding of individual subjects health and developmental conditions. Thus, we focused on the use of an uncooled thermoelectric infrared imaging sensor utilizing a thermopile focal plane array detector that features a 48 × 47 grid (2256 elements) made of silicon wafer and connected to the central processing unit of a computer by a serial peripheral interface. Because the sensing data unit was light enough, due to the low pixel image resolution, we could use higher time resolution, 1 Hz, to acquire data for one month, including both days and nights, in the home ambient condition. This allowed us to seek novel functions in the data [3]. The infrared sensor had been utilized not only in industry but also for human sensing [4]. Here we report our ability to acquire and process data on individual animals’ biological conditions and surrounding environment utilizing our low-cost, low-resolution, multiple index sensing system [5,6,7,8,9]. In particular, we focused on the infant neurodevelopmental stage as a time period that would feature common phasic shifts in locomotion and temperature, as well as in the proposed psychological index ‘place preference’. In addition to these index settings, our sensing system considered estimated environmental effects of room temperature average and gradient, and temperature and humidity outside the housing structure. Moreover, social environmental effects associated with feeding were assessed. Output from our inclusive sensor system design allows statistical analysis of developmental changes.

To find and validate valuable diagnostic functions and to secure longitudinal use of new sensor application technologies in humans demands preclinical trials in adequate animal models to avoid any risks both known and unknown.

Use of the common marmoset (*Callithrix jacchus*), a small New World monkey, as a biomedical model has increased dramatically in recent years. This increase has been driven by many factors including their phylogenetic proximity to humans, small size, high fecundity, rapid life history, and similarity to humans in social structure, behavior, cognition, communication and functional brain architecture, factors that make them a particularly good model of child health and human development.

Like other primates, marmosets recapitulate the core physiological properties and brain architecture of humans [10] and share ~93% sequence identity with the human genome (Marmoset Genome Sequencing Analysis Consortium, 2014). Common marmosets are reproductively competent by ~18 months of age, are monomorphic and reach an average adult body size of ~350–400 g by 2 years of age and at 8 years of age they are considered old [11,12,13]. Common marmoset gestation is 150 days and they generally produce twins or triplets every 5–6 months giving them among the highest fertility of any anthropoid primate [12]. The regularity of twinning in this species enables study designs that can effectively control for genetic and in utero contributions by using siblings in different study groups. Common marmosets live in human-like social groups, in general a pair-bonded male and female with their offspring, and cooperative care for offspring. This human-like social setting facilitates the study of social learning across generations [14]. Further validating the marmoset model of human development is their use of visual signals, including facial expression for communication [10,15] and their ability to imitate [16] which has only been reported previously in humans and chimpanzees. These similarities support the use of the common marmoset to model parenting and family effects on child development. While we appreciate that captive common marmosets live in a different environment than their wild counterparts, our subjects were born and bred in captivity over many generations and are adapted to their captive living environments making them an ideal model for studies of human development.

While the benefits of the marmoset model are clear, there are challenges associated with the use of this model. Of particular importance, behavioral assays routinely performed in popular animal models, rodents, have not been validated for use in common marmosets. One such tool for the assessment of neurodevelopment is a non-invasive, longitudinal method to evaluate an individual’s interaction with their social and physical environment through the monitoring of location and motion and associated circadian rhythms while in the home environment. Such assessments are of particular value given the relationship between early neurodevelopment and the development of circadian rhythms [17,18,19,20] with lifelong and potentially generational mental health [21,22,23] perhaps through epigenetic programming during the highly sensitive infant period [24,25,26].

In rodents, multiple methods are available for continuous monitoring of position and locomotion (e.g., [27,28,29,30]); however, in primates such model studies for humans to date have required instrumentation of animals [31] or could not be performed in the home environment [32]. Therefore, we developed a low-cost infrared (IR) technology-based location/motion, body surface temperature and surrounding ambient temperature detection system and analysis algorithm that can be used non-invasively for long-term studies of our primates in the home environment. We validated the use of this system by assessing early infant development in common marmosets, specifically the development of circadian rhythm, and propose the value of this technique for future studies of development as well as other studies for which continual non-invasive monitoring would be beneficial (e.g., neurodegenerative disease research). We further suggest the potential application of this technology to studies of human development as the information garnered from this technique may facilitate the development and implementation of programs designed to support healthy development. Thus, finally we attempted to verify the multivariate correlation cascade associated with developmental life events and environments on the complex biological circadian rhythm representation in a time-age manner.

## 2. Materials and Methods

### 2.1. Animals

This study was carried out in accordance with the recommendations in the Guide for the Care and Use of Laboratory Animals of the National Institutes of Health. All procedures were approved by the animal ethics review committee at the Tokyo University of Agriculture and Technology, TUAT (20–21). The studies described are all non-invasive and all efforts were made to minimize animal suffering.

Four common marmosets (*Callithrix jacchus*), two sets of twins (male/male and female/female), were purchased from a breeder (CLEA, JAPAN) at postnatal day (P)6 (males) or P8 (females). Upon arrival at Tokyo University of Agriculture and Technology and throughout the study period, animals were singly housed in a ventilated wooden and opaque box (1.0 w × 1.2 h × 0.9 dm) with a ceiling mounted IR thermal camera (TP-L0620EN, CHINO, JAPAN). For animals up to P31, an infantile plastic environment with a soft floor (Keiyo, Japan) placed above a sheet heater (Kyokko, Japan) was created within the home to regulate ambient temperature. At P31, when animals became able to regulate their body temperature, the environment was replaced with a steel environment and the sheet heater was removed. Experimenters’ parental handling was regarded as attachment care with warm handling and calling each name and benevolent words. Caring notebooks confirmed all the participants showed relax calling and affinity behaviors [21,33,34] to experimenters.

Marmosets were maintained on a 12:12 h lighting schedule with ambient temperature maintained according to an age-dependent schedule (P8–P14: 34 °C; P15–31: 32 °C; P32–P45: 30 °C). Animals were hand fed with a specified amount formula based on age, three times per day (morning, noon, night) until P29, and then twice per day (morning, night) from P30–P45. Each feeding period took approximately 30 min for gentle stimulus for defecation/urination and cleaning bodies with handling parental care. Each animal’s home was recorded before and after each feeding period. IR thermal camera recording was conducted continuously (except while animals were removed from the cage for feeding given the expected temperature and motion changes associated with the caregiver’s handling and feeding) from P15–P45 to assess animals’ location in the cage, motion and body temperature.

### 2.2. Image Processing and Data Acquisition

The ceiling-mounted IR camera continuously acquired thermal images (48 × 47 = 2256 pixels) of the floor and walls. Images were saved as csv files each second, and into directories each hour, as controlled by the application software TP-L02 (CHINO, JAPAN). The angle of field of the sensor we used was able to be selected as either 25 or 60 degrees depending on the specifications of the objective lens used. We chose the wider field lens (60 degrees) so the entire cage was included in the field of view. Our sensing technology was originally designed for automobiles and used filtered far-infrared light at 8 to 14 micrometers for thermally unique object detection with an accuracy of 0.5 °C and measurements taken three times per second. In order to automatically detect the XY position of a marmoset body we developed a new image-processing algorithm using Microsoft Visual C++ 2010 Express Edition (Microsoft, Japan) and OpenCV 2.2. IR-determined temperatures at each pixel of each image were converted into two-dimensional array variables. To adjust for growth, each day a square was manually drawn around the marmoset while sleeping to determine the area of the standard body size (S_base_). Background images were constructed by replacing the S_base_ with the temperature of the surrounding area and smoothing by 5 × 5 pixels.

Next, desired images were serially read and smoothed by 5 × 5 pixels following calculation of the standard deviation (SD). The background image to be subtracted from an image of interest was chosen based on the highest correlation coefficient (R_max_) with the desired image. In the image, by subtracting the background from the desired image following standardization and smoothing by 5 × 5 pixels of both images, a value less than zero in each pixel was regarded as zero and all the others were squared; then, the maximum value (MAX) was determined. The number of values more than MAX/2 (N1) and the area of the minimum square that could enclose all pixels more than MAX/2 (S1), the ratio of N1 to S1 (NS1), and the ratio of the vertical to the horizontal side of the square (VHratio) were calculated. Then, the images satisfying the following conditions were determined as parsable.

**Figure 1:**
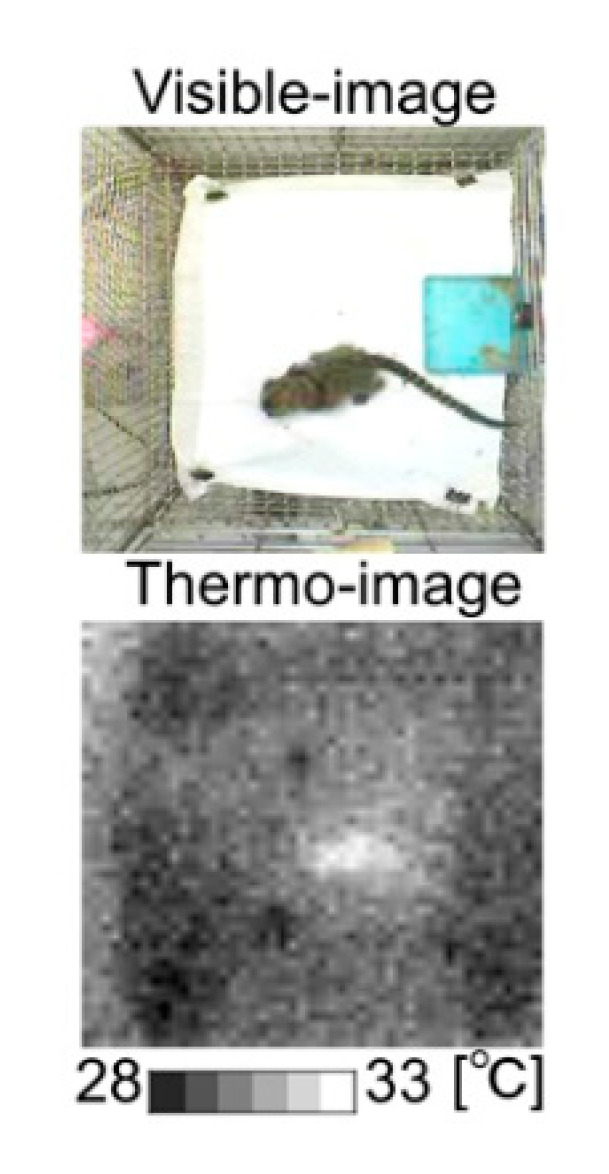
Example of common marmoset visible and thermal image.

1. S1 < S_base_ because the area of body size and the detected surface temperature increased independently of the actual surface temperature when the marmoset approached the camera.
2. NS1 > 0.6 to discriminate a marmoset from other objects based on their shapes, because the shape of a marmoset in the image was like a circle or ellipse (Figure 1), whereas those of other heat-generating elements were diagonally slender.
3. Rmax > 0.3 because the correlation coefficient became low when the pattern of the background was flat.
4. VHratio > 1/3 and <3 to distinguish a marmoset from a slender object, such as heat-generating elements.
5. MAX > 1.5 because when the temperature difference between a marmoset and the surroundings was too small, a false position was incorrectly detected as the marmoset’s position.
6. SD < 1.5 because when a marmoset approached the thermal camera too closely, the standard deviation became high due to the detection of increased temperature of the marmoset compared to the surrounding.

Because the thermal distribution of heat-map images varied based on the “ON” and “OFF” status of the heat generators, new background images were constantly generated from the parsable images. In the parsable images, the central XY position of the area enclosed by a square (S1) and its change per second were determined as the marmoset’s position and its biological locomotion velocity (BV), respectively. The maximum temperature in the vicinity of the central XY position (X ± 1, Y ± 1 pixel) was determined as the marmoset’s body-surface temperature (BT). The BV and BT were averaged every 30 min and corrected as follows:

1. Correction of BV. For BV, the difference between the distance in pixels and the actual distance depends on the distance between the camera and the marmoset. To normalize BV for changing height size, BV determined in the smaller height (up to P31) was multiplied by 320/700 (the height (mm) of the IR camera).
2. Correction of BT. We found preliminarily that the raw BT (BT_raw_) detected by the IR thermal camera fluctuated depending on the temperature inside the cage (IT). To remove the increment or decrement of the IT-dependent BT_raw_ signals, the model formula below was used, and BT_raw_ was normalized individually:
  BT = BT_raw_ − α * (IT − IT_ave_)
  BT: the corrected body-surface temperature, IT_ave_: the mean IT during the whole measurement period, alpha: the slope from the regression of BT_raw_ by IT.

### 2.3. Accuracy of the Developed Algorithm

To evaluate how precisely the XY position of a marmoset was detected by our image-processing method, test images were prepared from the analyzed images. Directories containing csv files for 1 h were numbered and a random number generator was used to select directories for analyses. The first 60 files in each selected directory were used for accuracy analysis. In total, 20 random numbers were generated in each age stage (St1–St4) for each individual, yielding 19,200 images that were used for the validation. Each image was visually inspected to determine if there was a marmoset present and if the square (S1) enclosed the marmoset. If true, the XY position, BV and BT were automatically obtained for each image. For all test images, the XY position at the center of the body, BV and BT were also manually extracted by visual inspection (ImageJ, 1.410, NIH, Bethesda, MD, USA).

We defined and calculated results for three indices for true and false determination for our image-processing method (Table 1). The algorithm invariably discriminated “no-marmoset images” as non-parsable and could discriminate 86% of “marmoset images” as parsable, with the lowest at St3 (74%). Almost 100% of parsable images were correctly parsable (Table 1). Approximately 14% of the images that could be analyzed were removed from the data set due to environmental noise. Linear regression comparing the automatic method we developed to manual data extraction confirmed that the methods were analogous (R^2^ were close to one (Table 2)).

Our results indicate that the image-processing algorithm we developed is both efficient and accurate, with almost 100% ability to correctly parse parsable images and a strong correlation with manual analysis techniques. These results further confirm that our image-processing method is reliable throughout the age range we tested and independent of both BV and ambient thermal condition.

### 2.4. Acquisition of Environmental Variables

Temperature inside the cage (IT, °C) was defined as the average of the temperatures from the four corners of the home cage. The accuracy of this measurement was evaluated by comparison between IT and temperature simultaneously logged by another thermo-recorder (MD6000 series, CHINO, JAPAN) every 5 min and averaged every 30 min. The two variables were highly correlated (R^2^ = 0.893) confirming the validity of IT. As another index of ambient temperature, we defined the mean value of the temperature in the two corners on the door-side of the cage minus the temperature in the two corners opposite the door side as an indoor temperature gradient toward the door (ID, °C) per second. IT and ID were averaged every 30 min.

Each animal’s home cage was enclosed and separated from the outside environment by four doors. Temperature within the cages was maintained at 28 °C with circulating filtered air. While this was designed to avoid the impact of outside air, the buildings outside temperature and humidity may have further influenced the marmosets’ development due to the challenges of consistently controlling IT (for example when either a room or cage door was opened even if the other three doors remained closed). The infant stayed inside the enclosed space while feeding with the second to fourth doors closed. To evaluate these potential effects, the outside temperature (OT) and relative humidity (OH) were obtained from the website of the Meteorological Agency in Japan (http://www.data.jma.go.jp/obd/stats/etrn/) every 30 min.

### 2.5. Definition of Biological Door Preference (BD)

Place preference has often been used as an estimate of motivation [35,36,37,38,39]. Here we defined time spent near the door of the cage (biological door preference [29]) as an indication of the animals’ motivation to explore outside of the home environment. To determine BD at the preliminary screening and to evaluate if a certain circadian periodicity emerged autonomously in double plots of the four subjects when averaging the data, while the XY position would rather reveal the common oscillation less in the individuals, we divided the floor of the home cage evenly into two areas: the door side and the opposite side and determined time spent in each half of the cage. If more than 50% of the time was spent on the door side of the cage this was considered as a preference for being near the door. An additional binary variable (BD (0.99)) was defined as “1” if the animal spent any time on the door side and “0” if the animal spent any time on the non-door side.

### 2.6. Data Extraction for ‘Feeding’-Related Analysis

Data for specific periods were extracted to assess animals at times during the light period but outside of feeding times as a strong estimate of its parental care effect and to compare information from around feeding times to other times during the light period or dark period. For each animal, data from 30-min blocks before and after each feeding period were identified as feeding. Additional 30-min blocks outside of these periods were identified as other-time. For animals up to P30, these included data for 30-min blocks both before and after the morning and evening feeding, and two successive 30-min blocks between both the morning and noon feeding (MN1, MN2), and noon and pm feeding (NE1, NE2). For animals P30 and older, sampling times remained the same with the exception of the elimination of the blocks before and after noon feeding as the noon feeding no longer occurred. For all animals, nighttime data were averaged in 2-h blocks.

### 2.7. Statistical Analyses

#### 2.7.1. Comparison between Light/Dark Periods and before/after Feeding

For multivariate analyses, data from animals throughout the developmental timeline (P15–45) were divided into four age stages based upon the development of a circadian locomotor pattern. Light-dark differences for each variable in each age stage were tested by two-way analysis of variance (ANOVA) with post hoc Tukey’s honestly significant difference (HSD) with factors for light/dark period and age stage or days of age. The response of each variable to feeding by age stage was similarly analyzed by two-way ANOVA followed by Tukey’s HSD with factors age stage and before/after feeding. Effect of feeding (before/after feeding) was also analyzed for each factor by a two-tailed, paired *t*-test.

Following our previously published methods (domestic chicks: [6,21,22,40,41,42,43]; common marmosets [6,7,8,9,21,33,34] and humans [6,21,44,45]), we then used principal component analysis (PCA) to infer the complex developmental mechanisms relating our obtained multiple variables including behavioral, physiological, biomolecular and psychological factors. The light-dark or before-after feeding differences of each principal component (PC) score were additionally analyzed by two-way ANOVA followed by Tukey’s HSD, as described above.

#### 2.7.2. Feeding-Dependent Explanatory/Response Variables

To compare the 4 age stages equally, the sample size at each stage was adjusted to six samples spaced as far away from each other as possible (St1: P15–P20, St2: P25–30, St3: P33–38, St4: P40–45). Before adopting the following approach of correlation model selection, we narrowed the number of nighttime data points to be assessed using Pearson correlation coefficients based on the assumption that nighttime memory formation [46,47] is affected by socio-emotional and physical experiences related to feeding. Each B variable of any paired time between feeding times and the focal night was explored, and the multivariate interactions considering the random effects among four marmosets were comprehensively evaluated in each stage (“intra-stages”) and across stages (“inter-stages”) based on linear mixed models (LMMs [48]) using R3.1.1 (The R Project for Statistical Computing, http://www.r-project.org/), with lme4 and cAIC4 packages. For “intra-stage” analysis, one of the B- variables before/after morning/evening feeding or at the focal night time was set as a response variable (‘R’), and fewer than four variables in seven B- (BV, BT, BD), I- (IT, ID), and O- (OT, OH) variables at the previous focal night times or before/after morning/evening feeding were set as explanatory variables (‘E’). The combinations of selected explanatory variables were _7_C_1_ + _7_C_2_ +_7_C_3_ + _7_C_4_ = 98 in one-explanatory model conditions. Both the fixed and random effects of B- and I- variables among 4 marmosets were considered in the regressions below, whereas no random effects were set for O-variables shared among them.
lmer(R~E1+(E1|ID))lmer(R~E1+E2+(E1+E2|ID))lmer(R~E1+E2+E3+(E1+E2+E3|ID))lmer(R~E1+E2+E3+E4+(E1+E2+E3+E4|ID))

For “inter-stage” analyses, the same variables used as “intra-stage” in the earlier and later age stage were set as ‘E’ and ‘R’, respectively, in a combination pair from St1–St4. In each paired-time condition, the best fitting model was selected based on cAIC (the conditional Akaike information criterion) [49]. To support the unstable estimation, models with the first to third lowest cAIC were selected as long as they allowed a difference of less than 1% from the first minimum cAIC. Once any absolute *t* value of these models was more than two, each of the estimated explanatory variables was used for the start of the following negative/positive correlation pathway vector whose end was the response. To specifically visualize responses to feeding, similar analyses were comparatively applied for the other times, MN1, MN2, NE1 and NE2, to replace the data around feeding and the specific explanatory response, which was set at each paired time to ‘feeding’ but not similar to the ‘other time’ at the formula level. Alternatively, an individual vector level was targeted. In contrast, the ‘other time’ or the ‘common’ points between ‘feeding’ and ‘other time’ specific vectors were independently collected. For the following analyses, the types of linear regression models were divided into subgroups of B-, I-, O-, BI, BO, IO, and BIO based on the combination of the explanatory variables. The ratios for each subgroup were calculated according to the total number of possible explanatory variables, 1680 in “intra-stages” and 7056 in “inter-stages” analyses. The ratios, except those left uncategorized, were analyzed by Pearson’s chi-square test followed by residual analysis to identify the ratios that were significantly different from all others.

The obtained pathways with the explanatory B-, I-, and O- variables as the start and response B-variables as the end per time and stage were summed. Consequently, connected networks appeared with convergence points (‘nodes’) by multiple pathways, which described not only the single relation but also diversified networks via relay nodes, and allowed realization of complex functions according to the effects of O- on B-indices or of B- on B-indices. For feeding-dependent pathways, any significant explanatory variable must be present in the data set from either before or after feeding time.

## 3. Results and Discussion

### 3.1. Definition of Age Stages

**Figure 2:**
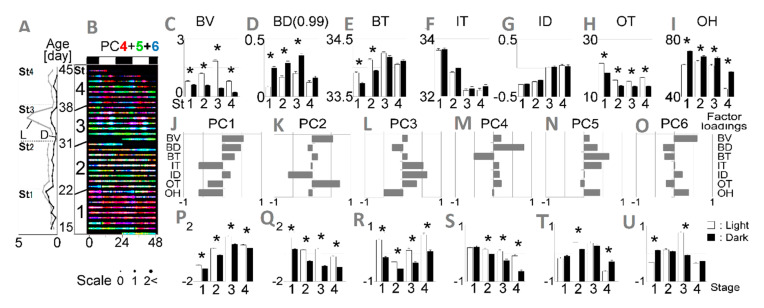
Developmental shift of circadian rhythm in three biological (B-) and four environmental (I-, O-) indices averaged in four infants and in terms of principal component scores. (**A**) Biological locomotion velocity (BV) trend lines compared between light (L) and dark (D) phases from age stage St1 to St4; (**B**) 30-min-raster plots (48 h horizontally versus postnatal day vertically) with a size marker at the bottom; overlaid raster plot of principal components PC4 (red), PC5 (green) and PC6 (blue). In C-I; Light-dark statistical comparison per age stage for each variable. Error bars indicate standard error of the mean (s.e.m.) * *p* < 0.05 by Tukey’s honestly significant difference (HSD) test following two-way analysis of variance (ANOVA); (**C**) biological locomotion velocity (BV); (**D**) place preference toward the door (BD) more than 99% (BD (0.99)); (**E**) body surface temperature (BT); (**F**) indoor temperature (IT); (**G**) indoor temperature gradient toward door (ID); (**H**) outdoor temperature (OT); (**I**) outdoor humidity (OH); (**J**–**O**) seven factor loadings of PC1–PC6. In (**P**–**U**) light-dark statistical comparison per age stage for PC1–PC6. Error bars indicate s.e.m. * *p* < 0.05 by Tukey’s HSD test following two-way ANOVA.

Age was segregated into four stages based upon the development of a circadian locomotor pattern as demonstrated by shifts in BV patterns (Figure 2A). P15–22 was defined as stage 1 (St1) during which time locomotor pattern differences between the light and dark phase were minimal. In stage 2 (St2; P23–31) distinct light-dark differences in BV were detectable as expected in this diurnal species [33]. In stage 3 (St3; P32–38) there was an obvious elevation in light-phase BV with minimal dark-phase BV and during stage 4 (St4; P39–45) light-phase BV was reduced and dark-phase BV remained minimal.

### 3.2. Testing of the Developed Algorithm through Evaluation of Age-Dependent Shifts in Circadian Rhythms

**Figure 3:**
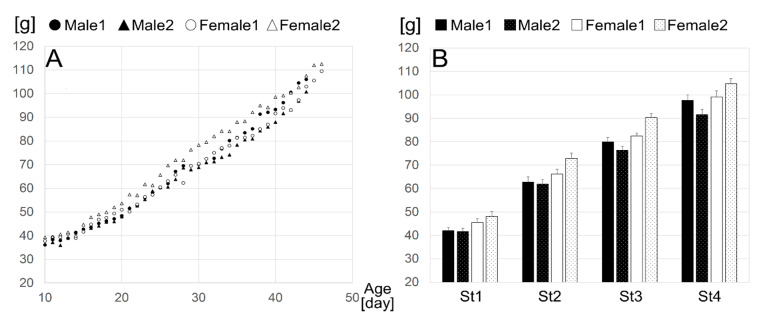
Longitudinal body weight. Body weight by age (**A**) and mean body weight by age stage (**B**). Age stages are St1:P15–22; St2:P23–31; St3:P32–38; St4:P39–45. Error bars indicate s.e.m.

All animals gained weight steadily throughout the study period (Figure 3) as appropriate, indicating that stress was not likely induced by the measurement procedure. The data from one-Hertz-rate raw IR images recorded over 30 days (P15–45) were post-processed, and trends in the biological (B-; BV, BD, BT) and indoor environmental (I-; IT, ID) indices were automatically captured. We first visualized the circadian rhythmicity of all seven variables (BV, BD, BT, IT, ID, OT, OH) by creating raster plots for each individual animal and the average of the four individuals (Figure 2). Day-night differences in each variable were evaluated to determine circadian rhythmicity (Figure 2).

As noted above, circadian rhythm of BV developed over time and was used to define the age stages used for analysis. BT, a physiological index, showed consistent circadian patterns in early age stages but varied in the late stage (Figure 2E). This finding confirms results from previous reports that diurnal body temperature rhythms emerge earlier than behavioral rhythms in primates and rodents [19,50,51] and are affected by behavioral [52] and thermal conditions [53]. BD was generally less phasic but BD (0.99) was more phasic (Figure 2D). Notably, nighttime BD (0.99) increased from St1 to St3 and then decreased at St4. Considering the understanding of place preference in experimental psychology [35,36,37,38], the BD age-dependent shifts could be interpreted as an index of the infants’ emotional development evoked by socially interactive feeding. There was a minimal relationship between BD and IT or ID (Figure 2F,G) suggesting a lack of preference based on internal environmental conditions. Both outdoor climate factors, OT (Figure 2H) and OH (Figure 2I), showed clear circadian rhythms which might impact development of circadian rhythms in B-indices even given the home cage temperature regulation. Consequently, we hypothesized that there was interplay among the seven variables, and we performed PCA with a correlation matrix to explore B-variables’ circadian interplay under weaker environmental influences.

In order to assess relationships among the seven variables, we performed a principal component analysis (PCA) with a correlation matrix to explore B-variables’ circadian interplay under weaker environmental influences. In seven acquired principal components (PC), the contribution rates were similar from PC1 (0.213) to PC6 (0.104) (Table 3). The factor loadings showed higher environmental influences in the PC1–3 (Figure 2J–L), and lower in the PC4–6 (Figure 2M–O). Thus, we visualized environment-independent development by superimposing the raster plots of PC4–6 (Figure 2B). This reconstruction identified novel circadian expression with age-dependent shifts, i.e., PC4 was dominant but less day/night phasic in St1. PC4–6 was unclear at St2, and PC6 was high during daytime in St3. Finally, significant alternate day-night rhythmicity emerged between PC4 and PC5 (Figure 2S–V).

### 3.3. Estimation of Developmental Cause and Effect Based on Socially Interactive Feeding

**Figure 4:**
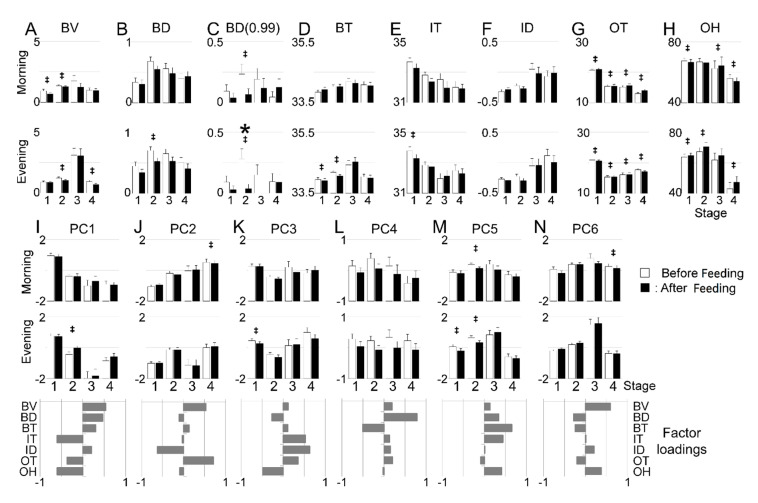
Comparison of each 30-min variable average before and after feeding. All variables (**A**–**H**) and PC scores characterized by factor loadings (**I**–**N**) are compared. * *p* < 0.05 by Tukey’s HSD test following two-way ANOVA. ‡ *p* < 0.05 by paired t-test.

The identified circadian rhythms were presumably driven by both innate and environmental factors including both the social and nutritive aspects of being hand-fed at a specific time each day. To identify any causal relationships during the age stages, we explored the eight variables’ correlations between two different time windows, i.e., before/after feeding or at night. The first comparison of each 30-min variable before and after feeding was summarized (Figure 4). Moderate differences were observed in the B- and O- indices, although only BD (0.99) at St2 in the evening was found to be significant by two-way ANOVA and Tukey’s HSD (Figure 4C).

**Figure 5:**
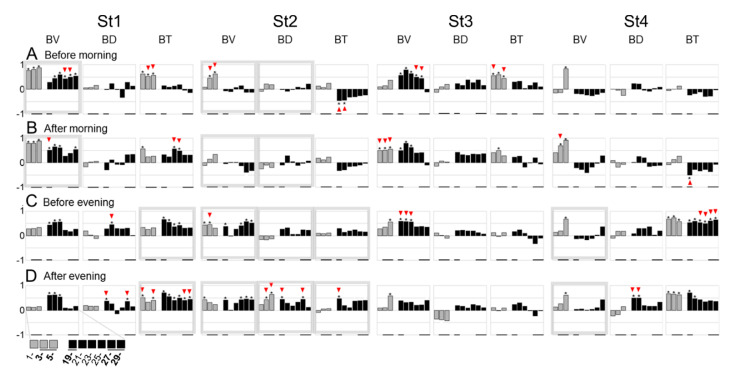
Screening of feeding dependence at night in two-hour increments on the basis of B- variables. Pearson correlation coefficients between the mean values 30 min before/after morning/evening feeding and in 2-h increments at night for each variable. * *p* < 0.05. Red triangle indicates differences before and after feeding in either ‘significant’ or ‘not significant’ results. A pair of graphs surrounded by a gray square denote significant differences before and after feeding. Black underbars indicate the focal night hours as determined by this screening.

**Figure 6:**
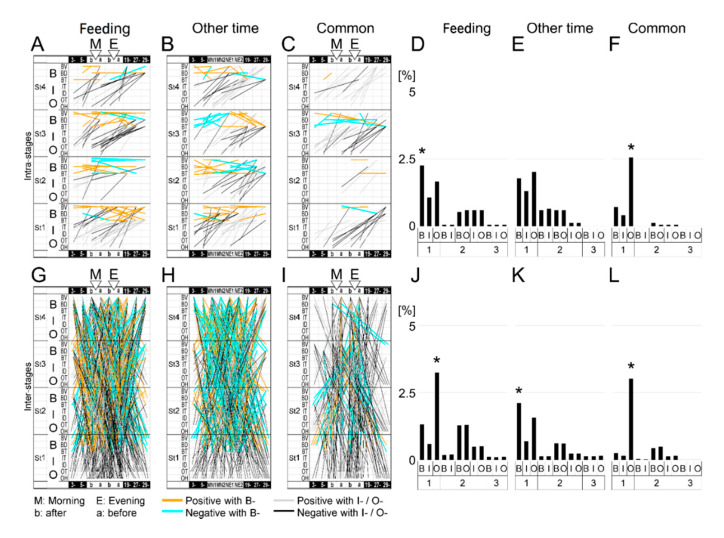
Correlation vectors among B-, I-, and O- variables of four age stages by linear mixed models (LMMs). Specific correlations to “feeding”, “other time” and “common” in the “intra-stage” analysis (**A**–**C**) and “inter-stage” analysis (**G**–**I**) based on LMMs followed by conditional Akaike information criterion (cAIC) to select the best fitting models are shown. Each vector indicates the relationship between an explanatory (earlier end in the time series) and response (later end in the time series) variable, and its color is categorized by the combination of the category of the variables, as shown at the bottom. Two or more vectors to one response variable from explanatory variables at same time and stage represent a complex explanation in one regression. The percentage of total explanatory variables’ number per single B-, I-, and O- indices or a combination of them are shown in each specific correlation pattern (**D**–**F**, **J**–**L**). * *p* < 0.05 by residual analysis following Pearson’s chi-square test.

**Figure 7:**
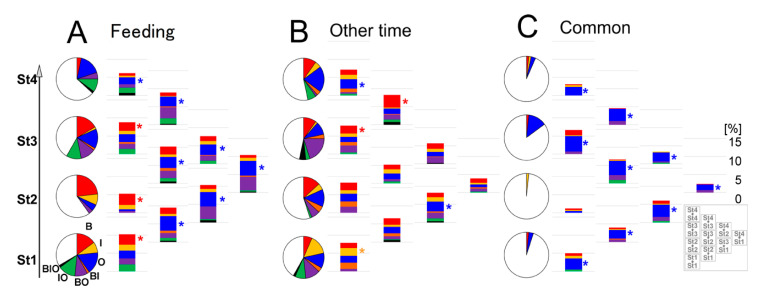
Correlated B-, I-, and O- variable ratios per intra- or inter-stage analysis by LMMs between two time points of feeding / other, day and night. In each specific condition of feeding (**A**), other day time (**B**), or common to both (**C**), pie charts represent the ratio of selected regression-model types, categorized as a single or combined contribution of significant B-, I-, O- variables based on LMMs followed by cAIC in “intra-stage” analysis in each age stage. The right bottom box represents each analyzed stage combination layout of the accumulated bars.

Next, the screening of feeding-dependent changes in B-variables in 2-h increments at night by Pearson’s correlation coefficient showed that correlated hours seemed to be generally sequential and continuous and were less correlated at midnight (Figure 5). Hence, to minimize the screening process, we used five representative nighttime periods (3- (3 to 5 o’clock), 5-, 19-, 27-(next day 3 to 5 o’clock), 29-). To analyze the relationships among before/after feeding and the five focal night periods, considering multivariate interactions, each B response variable was regressed on all B-, I-, and O- variables by use of linear mixed models (LMMs) [49] considering the random effects among the four individuals. These intra- and inter-stage analyses were further compared with the complementary analyses of day times other than feeding times and were categorized into three independent groups, ‘feeding, ‘other time’, and ‘common’ (Figure 6A–C,G–I). The obtained linear models were characterized by the explanatory variable combinations as B, I, O, BI, BO, IO, BIO. In Figure 7A, B- types were higher than other types in St1-3 but not St4, specifically in ‘feeding’, whereas O- types were more extensive than I- types in the inter-stages of ‘feeding’ and ‘common’ (Figure 7A,C). These findings suggest that longitudinally, but not acutely, climatic O- variables interacted with B- variables during feeding more than other times.

### 3.4. Feeding-Dependent B- Developmental Pathways of Intra- and Inter-Stages with Autonomic B- or Climatic O- Explanatory Variables

**Figure 8:**
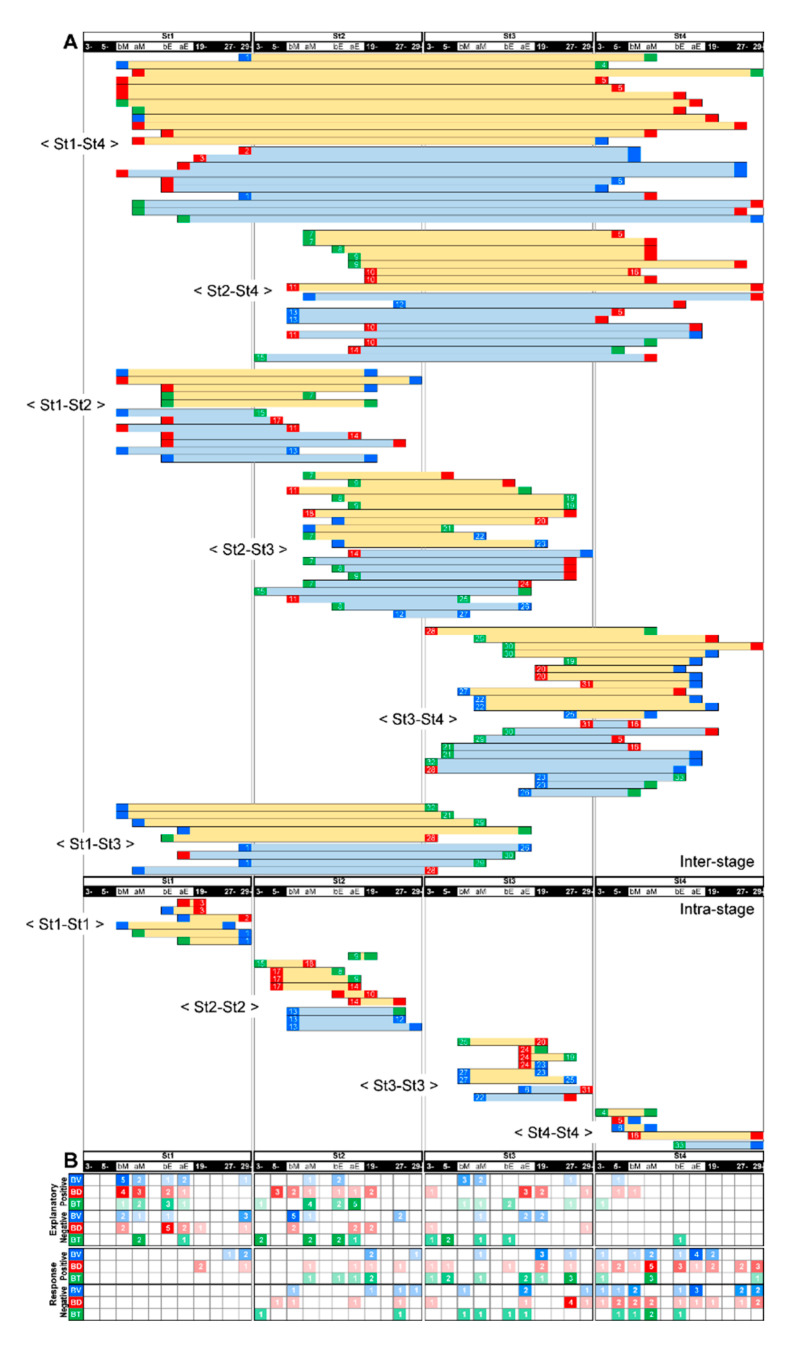
Feeding-dependent pathways of B-variables by LMMs. Age-stage and focal time are arranged horizontally in the time series. (**A**). Light yellow and light blue horizontal bars indicate positive and negative correlation vectors between explanatory (left side of the bars: start) and response (right side of the bars: end) variables, respectively. (**B**). The heat maps represent the summed number (color gradation) of positive or negative BV (V, blue), BD (D, red) and BT (T, green) as explanatory (*upper*) or response (*lower*) variables per focal time in each age-stage.

**Figure 9:**
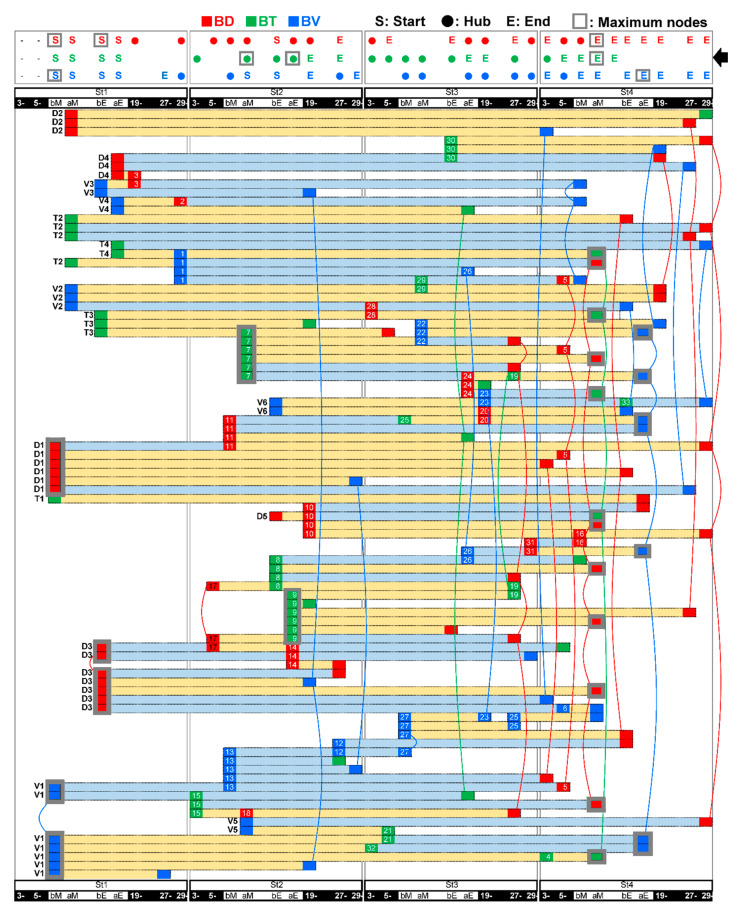
Connections of the feeding-dependent pathways depicted in Figure 8. The horizontal bars are the same as in Figure 8 re-arranged. The top table shows any presence of significant start (explanatory, “S”), hub-like relays (both explanatory and response, filled circle), or end (response, “E”) variables. Gray squares indicate the maximum response node (MRN) or maximum explanatory node (MEN) in each variable (see also Figure 10 and Figure 11). Same variables separately arranged are connected with one another with colored curves. The start points at St1 or St2 are marked by V1–V6, D1–D5 and T1–T4.

We explored how feeding-dependent B- development might explain the complex circadian shifts of PC4–6 (Figure 2B) by the B- or O- variables during the previous stage, as described in Figure 7. First, a pair of B-specific explanatory (start) and response (end) variables, which were present either before or after feeding, was represented as a vector over four stages (Figure 8). All vectors were connected to one another via any of the variables and times and were translatable as one complex developmental mechanism (Figure 8). Referring to B- explanatory and response variable numbers at each time point (Figure 8) or the presence/absence of a significant variable as ‘start’, ‘hub’, or ‘end’ (Figure 9, top table), the original start was estimated around feeding primarily in St1 and partially in St2. It was followed by the appearance of many ‘hub’ variables’ during St2–3; then, ‘end’ sequentially appeared in St4. Compared to other variables, the ‘end’ of BT significance uniquely disappeared during the late evening and night of St4 (Figure 9, black arrow), which might have contributed to the PCA-deduced circadian phases in St4 (Figure 2B).

**Figure 10:**
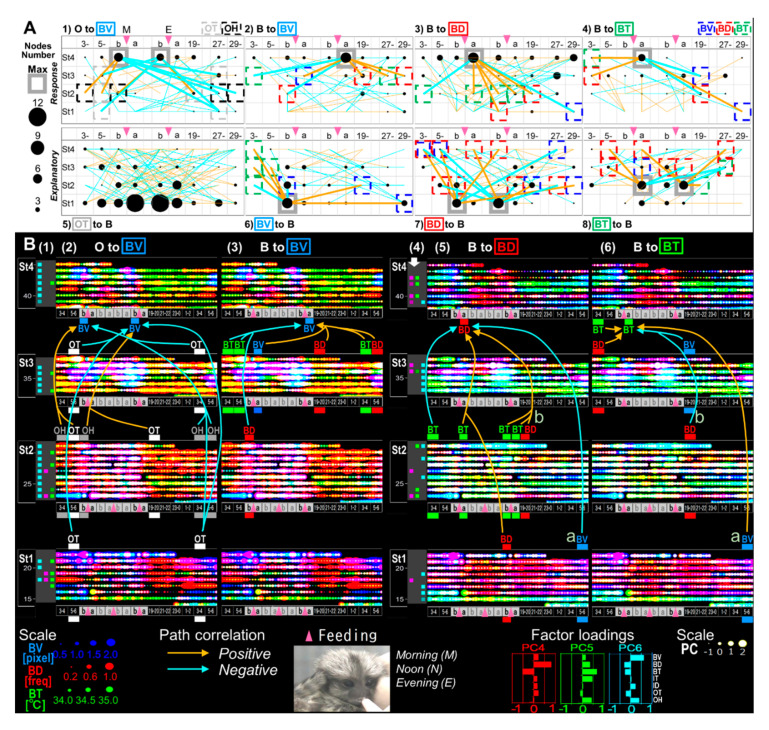
Feeding-dependent positive/negative pathways determined by LMMs on gradient or unique circadian development, on the basis of B(biological)-indices. (**A**) The numbers of explanatory or response variables (“nodes”) in each time point (horizontal axis; 3–4 (3-) and 5–6 (5-) a.m., before (b) and after (a) morning (M) and evening (E) feeding (pink arrow-head), 19–20 (19-) p.m., 27–28 (27-), 29–30 (29-) a.m.) are shown as circles, which estimate the effects of the variables. The effects as responses and explanatory variables are estimated by the regression of BV, BD or BT on arbitrary O- (1) or B- indices (2–4) and the regression of arbitrary B- indices on OT (5), BV, BD or BT (6–8; see also Figure 11). The maximum response nodes (MRNs) and maximum explanatory nodes (MENs) are marked by gray squares. (**B**) Overlaid 30-min-raster plot of BV (blue), BD (red) and BT (green; left side, 2–3) or PC4 (red), PC5 (green) and PC6 (blue; right side, 5–6), are displayed separately for each age stage. Significant light-dark differences in each day are shown as their own-colored squares on the left side (1), (4) of the raster plots (*p* < 0.05 (lighter colors) and *p* < 0.1 (darker colors) by Tukey’s HSD test following two-way ANOVA). The BV MRNs pathways regressed on O- indices (B2), “O to BV” (A1)), BV regressed on B- indices (B3), “B to BV” (A2)), BD regressed on B- indices (B5), “B to BD” (A3)), and BT regressed on B- indices (B6), “B to BT” (A4)) extracted by LMMs followed by cAIC are also depicted.

**Figure 11:**
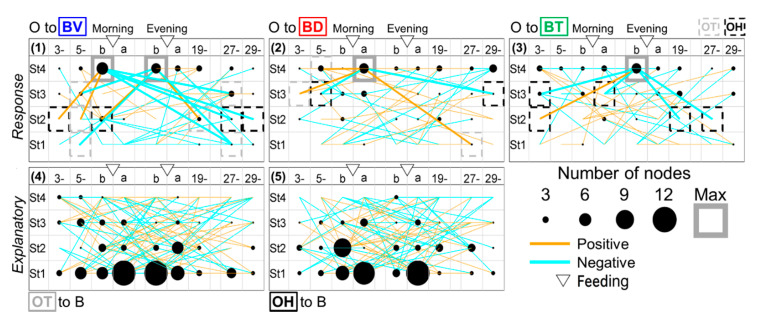
The number of nodes as explanatory or response in each time-point specifically in “feeding”. The numbers of explanatory or response variables (“nodes”) in each time-point are shown as the size of a filled circle, which can estimate the effects of the variables. The effects as response and explanatory are estimated by the regression of each B-variable on arbitrary O-indices (**1**–**3**), and the regression of arbitrary B-indices on each O-variable (**4**,**5**), respectively. The MRNs and MENs are marked by gray squares.

We further visualized which times might be described as nodes that influenced or were influenced by multiple factors in the intra-/inter-stage pathways by LMMs focusing on B (Figure 10(A2–A4,A6–A8)) and O- (Figure 10(A1,A5), Figure 11(1–5)) variables. O-variables showed the dominant number of projected pathways around feeding in St1, such as influential starts, diversified to multiple times over the age stages (Figure 11(4,5)). The maximum explanatory nodes (MENs) of BV (Figure 10(A6)) and BD (Figure 10(A7)) appeared at the time before feeding in St1. BV projected pathways to BT or BV at night in all age stages (Figure 10(A6)). BD directly and extensively influenced St4 (Figure 10(A7)), and BT MENs appeared after feeding at a different stage, St2 (Figure 10(A8)). This finding suggests that BT MENs might influence the following diversified pathways with a delay.

All of the maximum response nodes (MRNs; Figure 10(A1–A4), Figure 11 (1,2,3)) appeared around feeding in St4, irrespective of the explanatory variables. Regarding BV, a typical circadian variable, the MRNs after evening feeding in St4 were primarily pathways from St3 over day-night (Figure 10(A2)), which indicates that BV might be affected by the previous week. BD MRNs after morning feeding in St4 were explained by certain effects from St2 and St1 but not St3 with BT and BD factors (Figure 10(A3)). BT MRN after morning feeding in St4 may be explained by B-variables over age stages in nighttime but not daytime (Figure 10(A4)).

These results suggest that developmental ‘relay’ systems may be formed through complex psychophysical and circadian mechanisms via feeding experiences and nighttime biological activity. Because food and sociality are strong factors that induce anticipatory behavioral and physiological responses [54,55] as functions of the brain limbic systems [36,37,46,55,56,57], these differences in time-sequence before/after social and dietary rewards in developmental day–night cycles might reflect psychological memory of expectant and satisfied states.

### 3.5. A Trigger Candidate of the Feeding-Dependent B-Developmental Pathway Contributes to Circadian Biological Door Preference-Body Surface Temperature (BD-BT) Correlation Switching

**Figure 12:**
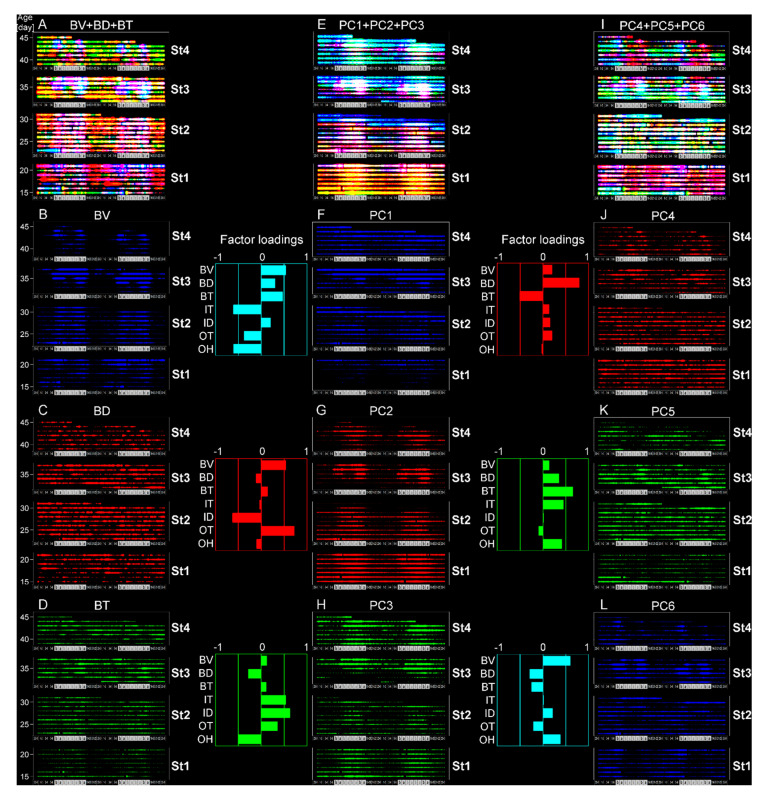
Characterization of complex circadian rhythms. Overlaid raster plots of BV ((**B**), blue), BD ((**C**), red) and BT ((**D**), green), or PC1 ((**F**), blue), PC2 ((**G**), red) and PC3 ((**H**), green), or PC4 ((**J**), red), PC5 ((**K**), green) and PC6 ((**L**), blue) are depicted in (**A**), (**E**) or (**I**), respectively.

Finally, to extrapolate feeding-dependent developmental mechanisms under the influence of the interplay of multiple factors [58,59], the pathways of the B-MRNs from O-/B- were represented as two types of 3 color-superimposed plots (Figure 10B). One is from B- indices (Figure 10(B2,B3), Figure 12A–D), and another is from PC4–6 (Figure 10(B5,B6), Figure 12I–L) to reduce either O-/I-effects (Figure 12E–H) based on the factor loadings (Figure 2J–O). The former results may have involved interplay between the dominant O- and BV in the B- variables (Figure 10(B2,B3)), which ambiguously expressed color gradient shifting as red in St1, orange in St2, yellow in St3, and green in St4. Hence, the alteration of Figure 10(B2,B3) is explained as ‘flat shifting’ with a less critical change, except in the BV circadian phases (two-way ANOVA in Figure 10(B1)). Thus, we added the BV-relevant LMM pathways from either O- or B- explanatory variables with the MRNs in St4 (Figure 10(A1–4)) and then compared them above the B-variable plots (Figure 10(B2,B3)). The pathway from B- to BV (Figure 10(A2)) was primarily projected from St3 (Figure 10(B3)), whereas the pathway from O- to BV (Figure 10(A1)) was frequently shown to be longer from St1–3 (Figure 10(B2)), consistently with the higher O- ratios in the inter-stage than the intra-stage (Figure 7A).

**Figure 13:**
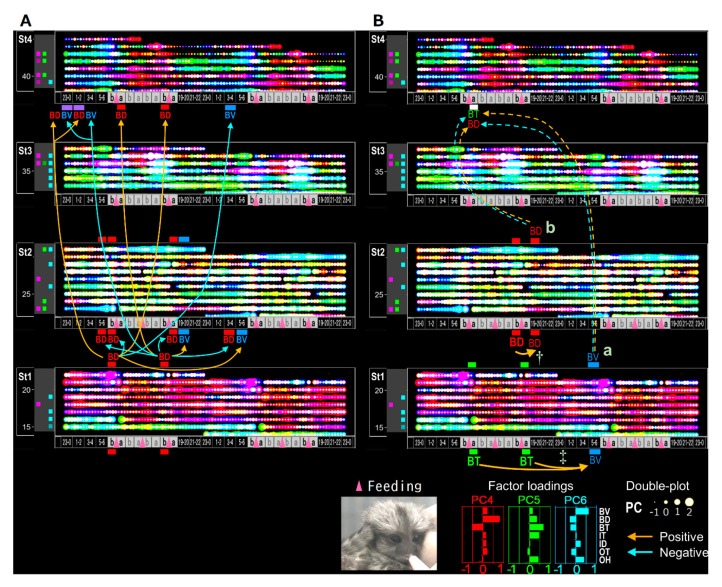
The origin of candidate trigger pathways revealed in Figure 10. The double plots are same as shown in Figure 10B, overlay of PC4–PC6. (**A**) The pathways from the maximum explanatory nodes revealed in the regression of arbitrary B-indices on BD. (**B**) Solid lines marked by a dagger and double dagger indicate the original pathways of key pathways marked by “a” and “b” corresponding to “a” and “b” in Figure 10(B5,B6).

Last, we compared the relevant B- to BD (Figure 10(A3) and B- to BT (Figure 10(A4)) pathways to the circadian BD-BT pattern, particularly in St4 (Figure 10(B4) (white arrow)) visualized by PC4–6 overlay (Figure 10(B5,B6)). Both MRNs emerged after morning feeding when infants were sated. Their behavior after feeding was different from than before feeding, presumably due to different expectations associated with the caregiver. Following morning feeding in St.4, we found two notable oppositely paired positive/negative correlating branches from the same night times and explanatory variables but different response-variable sets. One pair started BV from 5–6 a.m. as the night before morning in St1 (Figure 10(B5,B6,Ba)) and another, BD from 7 p.m. as the night beginning in St2 (Figure 10(B5,B6,Ba).The common MRN of BD and BT in St4 was consistently around the beginning of the PC4 (red) dominant phase because of the negative BD-BT correlation of PC4. This key pathway origin was confirmed in BT after morning/evening feeding in St1 and BD before evening feeding in St2 (Figure 13B, daggers), which might explain the effect of the previously noted difference in BD and BT around feeding in Figure 4B–D (evening) as trigger candidates for late-stage emotional circadian development in St4. Additionally, considering the presence of a simpler straight pathway from BD before evening feeding in St1 toward after morning feeding in St4 (Figure 13A), both pathways suggested that each psychobiological character either before or after feeding was independent in the early stage (St1–2) but converged later (St4). The former pathway passed circuits via different B-mechanisms and then reached St4 (Figure 13B). The circadian positive and negative correlation shift emerged (Figure 2B) as contributing to the formation of more complex emotional mechanisms with a psychologically relevant BD [35,36,37,38] –BT [57,60] correlation. In contrast, the latter influenced the underlying basic mechanism because of the longitudinal effect in St2 but not St3, just after the dramatic change in home environment and when feeding times were reduced from three to two.

It is reasonable to assume that feeding has among the strongest impact on infantile development. The current correlation analysis of marmosets before and after feeding by LMMs might allow us to decipher a certain “learning” in both intra- and inter-stage comparisons. This approach has the potential to reveal not only individual responses but also species-specific development or evolutionary mechanisms. Our data-driven comprehensive visualization approaches might reveal cause and effect estimations of interactive developmental processes although further studies are required.

This biological and environmental measurement system was designed to use a low-cost IR image sensor that also required lower data-processing costs providing general versatility to apply this technique to human studies however, as a trade-off, the detection ability was limited. More recently available high-end IR image sensors and big data processing technology must be used to further visualize novel developmental neurobiology.

## 4. Conclusions

We have shown the validation and application of an inclusive sensing technique for the continuous non-invasive collection and assessment of home position, body temperature, locomotion and environmental 1 Hz data for one month using an inexpensive IR thermal sensor in a primate model. Through validation of this methodology, we have uncovered new information about the common development of circadian rhythm in four common marmosets and its relationship to ambient and climatic, social and biological variables. We consistently detected common developmental cascades and multiple factors in time-age nodes appeared as estimated causes and responses in the network structures. Limitations of the study include the animals’ living situation. While we have shown this technique to be particularly effective in singly housed primates, it could be readily adapted for human use at home with the addition of non-invasive IR or wireless tags. The potential application of this sensor technology to humans may facilitate the development and implementation of home artificial intelligence programs designed to support healthy development.

## Figures and Tables

**Table 1:** Accuracy of the image-processing method.

	Index	All	St1	St2	St3	St4
1	The ratio of non-parsable images in the images in which there was no marmoset	1.000	1.000	1.000	1.000	1.000
2	The ratio of parsable images in the images in which there was a marmoset	0.858	0.919	0.943	0.740	0.831
3	The ratio of correctly parsable images in all the parsable images	0.998	0.998	0.996	1.000	0.999

**Table 2:** Slope (alpha) and correlation coefficients (R^2^) from the regression of values extracted by the image-processing method and manual analysis without intercept. (manual data) = alpha x (automatic data).

		All	St1	St2	St3	St4
alpha	X	0.998	0.993	0.995	1.004	1.003
	Y	1.020	1.027	1.033	1.010	1.011
	BV	1.038	1.052	1.073	0.966	0.965
	BT	0.999	0.999	0.999	1.001	0.999
R^2^	X	0.992	0.992	0.989	0.997	0.996
	Y	0.987	0.990	0.976	0.991	0.995
	BV	0.964	0.965	0.961	0.968	0.968
	BT	0.935	0.919	0.911	0.962	0.966

**Table 3:** Contribution rates of principal component analysis (PCA) with seven variables.

	PC1	PC2	PC3	PC4	PC5	PC6	PC7
Contribution Rate	0.213	0.174	0.163	0.143	0.132	0.104	0.072
Cumulative Contribution Ratio	0.213	0.386	0.550	0.692	0.824	0.928	1.000
